# Evaluation of Long-Time Decoction-Detoxicated *Hei-Shun-Pian* (Processed *Aconitum carmichaeli* Debeaux Lateral Root With Peel) for Its Acute Toxicity and Therapeutic Effect on Mono-Iodoacetate Induced Osteoarthritis

**DOI:** 10.3389/fphar.2020.01053

**Published:** 2020-07-24

**Authors:** Lei Zhang, Ting Li, Rongrong Wang, Jiaan Xu, Li Zhou, Li Yan, Zhengyan Hu, Hongwen Li, Fucun Liu, Wenxi Du, Peijian Tong, Huiling Wu, Shanxing Zhang, Letian Shan, Thomas Efferth

**Affiliations:** ^1^ School of Biological and Chemical Engineering, Zhejiang University of Science and Technology, Hangzhou, China; ^2^ The First Affiliated Hospital, Zhejiang University School of Medicine, Hangzhou, China; ^3^ College of Pharmacy, Zhejiang Chinese Medical University, Hangzhou, China; ^4^ The First Affiliated Hospital, Zhejiang Chinese Medical University, Hangzhou, China; ^5^ Department of Physicochemistry and Toxicology, Center for Disease Control and Prevention of Zhejiang Province, Hangzhou, China; ^6^ Experimental and Training Center, Zhejiang Pharmaceutical College, Ningbo, China; ^7^ Department of Orthopaedics, Changzheng Hospital, Second Military Medical University, Shanghai, China; ^8^ Department of Pharmaceutical Biology, Institute of Pharmacy and Biochemistry, Johannes Gutenberg University, Mainz, Germany

**Keywords:** *Aconitum carmichaeli*, traditional Chinese medicine, osteoarthritis, monosodium iodoacetate, pain behaviour, analgesia

## Abstract

**Background:**

As a degenerative joint disease with severe cartilage destruction and pain, osteoarthritis (OA) has no satisfactory therapy to date. In traditional Chinese medicine (TCM), *Aconitum carmichaeli* Debeaux derived *Hei-shun-pian* (*Hsp*) has been developed for joint pain treatment. However, it causes adverse events in OA patients. Long-time decoction has been traditionally applied to reduce the aconite toxicity of *Hsp* and other aconite herbs, but its detoxifying effect is uncertain.

**Methods:**

*Hsp* was extracted with dilute decoction times (30, 60, and 120 min) and evaluated by toxicological, chemical, pharmacological assays. Acute toxicity assay and chemical analysis were employed to determine the toxicity and chemoprofile of *Hsp* extracts, respectively. Since the detoxified *Hsp* (d*Hsp*) was defined, its therapeutic effect was evaluated by using an OA rat model induced by monosodium iodoacetate. d*Hsp* at 14 g/kg was orally administered for 28 days, and the pain assessments (mechanical withdrawal threshold and thermal withdrawal latency) and histopathological analyses (HE and safranin-O staining) were performed. Real-time PCR (qPCR) was applied to determine the molecular actions of d*Hsp* on cartilage tissue and on chondrocytes. MTT assay was conducted to evaluate the effect of d*Hsp* on the cell viability of chondrocytes. The cellular and molecular assays were also conducted to analyze the functions of chemical components in d*Hsp*.

**Results:**

The chemoprofile result showed that the contents of toxic alkaloids (aconitine, mesaconitine, and hypaconitine) were decreased but that of non-toxic alkaloids (benzoylaconitine, benzoylmesaconitine, and benzoylhypaconitine) were increased with increasing decoction time. Acute toxicity assay showed that only *Hsp* extract with 120 min decoction was non-toxic within the therapeutic dose range. Thus, it was defined as d*Hsp* for further experiment. In OA experiment, d*Hsp* significantly attenuated joint pain and prevented articular degeneration from MIA attack. qPCR data showed that d*Hsp* restored the abnormal expressions of *Col10*, *Mmp2*, *Sox5*, *Adamts4/5/9*, and up-regulated *Col2* expression in rat cartilage. *In vitro*, d*Hsp-*containing serum significantly proliferated rat chondrocytes and regulated the gene expressions of *Col2*, *Mmp1*, *Adamts9*, and *Aggrecan* in a similar way as the *in vivo* data. Moreover, aconitine, mesaconitine, and hypaconitine exerted cytotoxic effects on chondrocytes, while benzoylaconitine and benzoylhypaconitine except benzoylmesaconitine exhibited similar molecular actions to d*Hsp*, indicating contributions of benzoylaconitine and benzoylhypaconitine to d*Hsp*.

**Conclusions:**

This study defined d*Hsp* and demonstrated d*Hsp* as a potential analgesic and disease modifying agent against OA with molecular actions on the suppression of chondrocyte hypertrophy and extracellular matrix degradation, providing a promising TCM candidate for OA therapy.

## Introduction 

Osteoarthritis (OA) is a progressive joint disease characterized by cartilage degradation, sclerosis of subchondral bone and osteophyte formation, resulting in chronic joint pain, joint stiffness, and disability. OA is the main cause of lower extremity disability around the globe, with hip and knee OA accounting for 17 million years lived with disability or 2.2% of all-cause years lived with disability (O’Neill et al., 2018). The incidence and severity of OA in women are higher than that in men. It is estimated that the lifetime risk of knee OA is about 40% in men and 47% in women (Johnson and Hunter, 2014). Even worse, due to the aging of the global population and the aggravation of obesity, the incidence of OA is getting higher and is expected to double by the year 2020 (Johnson and Hunter, 2014; Mandl, 2019). Current OA treatments are mostly targeting the symptomatic relief of pain and inflammation for joint function improvement. However, none of them can modify the OA progression, and their therapeutic outcomes are often associated with incomplete relief and side effects (Bijlsma et al., 2011). Therefore, development of novel anti-OA therapeutics is still sorely needed.

The lateral root of *Aconitum carmichaeli* Debx (family Ranunculaceae), named *Fu-zi* in China, is a widely used traditional Chinese medicine (TCM) with cardiotonic, analgesic, and anti-inflammatory activities. It was originally described by the earliest Pharmacopeia of China, “*Shennong Materia Medica*” (24−220 AD). However, *Fu-zi* is highly toxic, and diester-diterpenoid alkaloids (DDAs) such as aconitine, mesaconitine and hypaconitine are its main toxic components (He et al., 2017). These components can cause toxic side effects on the cardiovascular, nervous, respiratory and digestive systems, which can be mainly manifested as arrhythmia, hypotension, hypothermia, respiratory depression, muscle paralysis and central nervous dysfunction, and may even lead to death in severe cases (Chan, 2009). These side effects limited the clinical application of *Fu-zi*. Traditionally, processing methods have been developed for reduction of *Fu-zi*′s toxicity prior to prescription (China Pharmacopeia Committee, 2015). *Hei-shun-pian* (*Hsp*) is such a processed product that has been widely used as a principal herb in TCM formulas for treatment of joint pain, including *Gan-cao Fu-zi Tang* and *Fu-zi Tang* (Huang, 2013). Modern clinical studies have reported the therapeutic effects of those formulas on knee OA (Deng, 2008). Recently, we also reported in a clinical trial that *Fu-zi Tang* effectively alleviated knee pain and improved life quality of patients with mild to moderate knee OA (Liu et al., 2016). Nevertheless, adverse events still occurred with *Fu-zi Tang* treatment in those patients, indicating that *Hsp* remained toxic. It is well known that long-time decoction is a traditional and useful processing method for detoxifying aconite toxicity, resulting in derivatization of non-toxic alkaloids (benzoylaconitine, benzoylmesaconitine, and benzoylhypaconitine) from toxic alkaloids (Tong et al., 2013). However, there is no standard procedure to optimize detoxification, resulting in potential risks for patients. To evaluate the traditional detoxifying effect on *Hsp* and to explore, whether or not the long-time decocted *Hsp* remains therapeutically effective, we conducted acute toxicity assay and chemical analysis to determine the detoxicated *Hsp* (d*Hsp*) and then employed an OA rat model to evaluate the therapeutic effect of d*Hsp*. Afterwards, the molecular actions of d*Hsp* on cartilage tissue and chondrocytes were clarified by real time PCR. This is the first report regarding the development and evaluation of d*Hsp*.

## Materials and Methods

### Preparation of *Hsp* Extracts

*Hei-shun-pian*, a processed product of the lateral root of *Aconitum carmichaeli* Debx (Ranunculaceae) was harvested from Jiangyou (Sichuan, China) and authenticated by the authors (voucher specimen No. 081102). Detoxifying processing was applied in accordance with the procedure described in our previous report (Tong et al., 2013). The specific steps are as follows: The materials were powdered and evenly divided into three samples. Each sample was soaked in 10-fold water for 30 min, followed by boiling and decocting for 30 min, 60 min, and 120 min, respectively. Then, the water extract of each sample was collected after filtration, and 8-fold the amount of water was added to each residue, which is the same as the above procedure to decoct again, and to combine the first-stage extracts with the second-stage extracts of each sample. Finally, each supernatant was concentrated by rotary evaporation and freeze-dried to powder for storage and diluted into 1.0 g/ml for use. The extracts with 30 min decoction, 60 min decoction, 120 min decoction were labeled as *Hsp*-30, *Hsp*-60, and *Hsp*-120, respectively.

### Chemicals and Reagents

Standard substances (HPLC grade) of aconitine (98.01% of purity, batch number: MUST-19110905), mesaconitine (99.14% of purity, batch number: MUST-19111311) and hypaconitine (99.09% of purity, batch number: MUST-19080210), benzoylaconitine (99.44% of purity, batch number: MUST-19103010), benzoylmesaconitine (99.66% of purity, batch number: MUST-19032807), and benzoylhypaconitine (99.46% of purity, batch number: MUST-20022710) were purchased from Chengdu Must Bioscience and Technology CO., LTD (Chengdu, China). Methanol and acetonitrile were of HPLC grade (Tedia, Fairfield, USA). Ammonium acetate and tetrahydrofuran were of analytical grade. Mono-iodoacetate (MIA) was purchased from Sigma (St. Louis, MO, United States). Iscove’s modified Dulbecco’s medium (IMDM), fetal bovine serum (FBS) and 0.25% trypsin were obtained from Gibco (Thermo Fisher Scientific, Inc., Waltham, MA, United States). 3-(4, 5-Dimethylthiazol-2-yl)-2,5-diphenyltetrazolium bromide (MTT) and dimethyl sulfoxide (DMSO) were obtained from Sigma-Aldrich (Taufkirchen, Germany). TRIzol reagent was purchased from Thermo Fisher Scientific Inc. The real time polymerase chain reaction (PCR) kit was purchased from Takara Biotechnology Co., Ltd. (Dalian, China). All antibodies were purchased from Cell Signaling Technology, Inc. (Danvers, MA, United States).

### Animals and Cell Line

Male SD rats (180–220 g) and Kunming mice with both sexes (18–22 g) were purchased from Shanghai Laboratory Animal Center of Chinese Academy of Sciences (Grade SPF II Certificate No. SCXK2017-0005) and housed in ventilated cages at 22 ± 1°C under a 12/12 h light/dark cycle with water and food *ad libitum*. Primary chondrocyte was isolated from the rat articular cartilage and cultured as previously described (Wang et al., 2013).

### Chemical Analysis

HPLC analysis was performed on an Agilent 1260 Infinity HPLC system (Agilent Technologies, CA, United States). Chromatographic separation was carried out on a Hypersil BDS C_18_ column (250×4.6 mm, 5 μm) (Dalian Elite Analytical Instrument Co., Ltd) at 30°C. The mobile phase consisted of 0.1 mol/l ammonium acetate solution and acetonitrile-tetrahydrofuran (100:50, v/v) with a flow rate of 1.0 ml/min. The elution gradient was started at 85% ammonium acetate solution, followed by decreasing the ammonium acetate solution to 74% within 40 min, subsequently, the mobile phase was switched to 85% ammonium acetate again for 45 min. The sample injection volume was 10 μl, and the detection wavelength was 235 nm. The standard substances of aconitine, mesaconitine and hypaconitine were dissolved in methanol to obtain working standard solutions. The data was analyzed to identify and quantify the aconitine, mesaconitine, and hypaconitine in *Hsp*-30, *Hsp*-60, and *Hsp*-120 extracts by two-point external standard method.

The UPLC-MS analysis was performed on an Acquity UPLC system (Waters, MA, USA) equipped with a Xevo TQ-S triple quadrupoleelectrospray ionization (ESI) MS (Waters, MA, USA) operated in positive ESI-mode. Chromatographic separation was carried out on an Acquity BEH C_18_ column (100 mm × 2.1 mm, particle size 1.7 μm) maintained at 40°C. The mobile phase consisted of 5 mM ammonium formate solution (0.1% formic acid) and methyl alcohol (55:45, v/v) with a flow rate of 0.4 ml/min. The sample injection volume was 5 μl. The standard substances of benzoylaconitine, benzoylmesaconitine and benzoylhypaconitine were dissolved in methyl alcohol to obtain working standard solutions. The data was analyzed to identify and quantify the benzoylaconitine, benzoylmesaconitine, and benzoylhypaconitine in *Hsp*-30, *Hsp*-60, and *Hsp*-120 extracts.

### Acute Toxicity Assay

Male and female Kunming mice were randomly divided into 16 groups with 8 animals each (4 male and 4 female). Before oral administration, all mice were fasted for 12 h with water *ad libitum*. Each five groups were orally given one type of detoxicated *Hsp* (*Hsp*-30, *Hsp*-60, or *Hsp*-120) in doses of 40, 60, 80, 100, 120 g/kg, respectively. The control group received an equal volume of water. After a single administration, the animals were observed closely during first 24 h and were kept under observation up to 14 days. The mortality of each group was observed. As an index for acute toxicity, LD_50_ with associated 95% confidence limits (CL) was determined by the Bliss’s method.

### Therapeutic Evaluation

A total of 30 rats were used for therapeutic evaluation of d*Hsp*. All rats were randomly and equally divided into three groups: NC as normal control group, OA as model group, and OA+d*Hsp* as d*Hsp* treated OA group. The OA and OA+d*Hsp* groups were intra-articularly injected with 50 μl of 20 mg/ml MIA through the patella ligament of rat knees using a 100 μl microliter syringe to establish the OA model, while NC group was treated with 50 μl of saline in a same way. Besides the MIA injection, rats in OA+d*Hsp* group were simultaneously treated with oral administration of 14 g/kg d*Hsp*, while OA and NC group were given the equal volume of saline, respectively. The treatment lasted for 28 days, and the pain-related mechanical withdrawal threshold (MWT) and thermal withdrawal latency (TWL) were tested after the last treatment. In addition, the serum of rats from NC group and OA+d*Hsp* group were collected to obtain blank serum and d*Hsp-*containing serum for *in vitro* experiment. Finally, all the animals were sacrificed under anesthesia, and the knee joints were taken immediately for histopathological and real time PCR assay.

### Pain-Related Behavioral Observation

The TWL and MWT were measured by a Plantar Test apparatus (UgoBasile, Italy) and the von Frey test, respectively. The rats were placed in a transparent plastic box at room temperature (25 ± 2) °C. After the rats were quiet (stop combing hair and exploring activities), the cross mark on the tester was placed in the center of the left rear sole of the rats, and the instrument was opened away from the foot pad. Each rat was measured three times. In order to prevent the rats from being scalded by thermal radiation, the upper limit of time and temperature was set as 20 s and 35°C, respectively. For testing the mechanical pain threshold, the von Frey needle of the instrument was used to press the plantar surface of the left and right hind paws of each rat about three times. The rats showed rapid withdrawal of claws and licking of claws as the positive reaction of each test.

### Histopathological Observation

The rat joints on one side were dissected immediately after sacrifice and fixed in 4% paraformaldehyde for 48 h, followed by decalcification with 5% hydrochloric acid for 96 h. After paraffin embedding, samples were sectioned (4~5 μm) and stained with HE (hematoxylin and eosin) and SO (safranin-O) using routine process. The HE stained slides were photographed under the microscope. According to Mankin’s scoring system, statistical grading was carried out in the range of 0–13 points through double-blind observation.

### Cell Viability Assay

The chondrocyte viability was determined by MTT assay. The 2nd generation of chondrocytes were seeded on 96-well plates at a density of 5×10^3^ cells/well in 200 ml medium for 24 h, and treated with d*Hsp-*containing serum at 2.5, 5, 10, 15% for 24, 48, and 72 h and then treated with aconitine, mesaconitine, hypaconitine, benzoylaconitine, benzoylmesaconitine, and benzoylhypaconitine at dose ranges according to their concentrations in *Hsp*-120. A total of 20 ml MTT solution (5.0 mg/ml) was added to each well and incubated at 37°C for 4 h. 150 ml DMSO was subsequently added to each well to dissolve the formazan crystals and the optical density (OD) value was measured at 490 nm with a microplate reader (Bio-Rad Laboratories, Inc., Hercules, CA, United States). Proliferative rate (%) = (d*Hsp*-treated OD/untreated OD) ×100.

### Real-Time PCR (qPCR)

qPCR assay was performed to test the relative mRNA expression of OA-related genes of rat joints obtained from the animal experiment and chondrocytes by using an ABI QuantStudio™ 7 Flex Real-Time PCR System (Applied Biosystems; Thermo Fisher Scientific, Inc.). An aliquot of chondrocytes were pre-treated with TNF-α (10 ng/ml) for 6 h, and then treated with d*Hsp*-containing serum (15%) for 24 h. Another aliquot of chondrocytes were treated with benzoylaconitine, benzoylmesaconitine and benzoylhypaconitine for 24 h at doses corresponding to their concentrations in *Hsp*-120. Total RNA was extracted from cartilage tissue or chondrocytes using TRIzol reagent and reverse transcription was performed to produce cDNA. The final PCR reaction system was 20.0 μl, comprising 10.0 μl SYBR^®^ Premix Ex Taq II (Tli RnaseH Plus), 0.8 μl PCR Forward Primer, 0.8 μl PCR Reverse Primer, 2.0 μl template cDNA, 0.4 μl ROX Reference Dye, and 6.0 μl ddH_2_O. The qPCR reaction conditions were as follows: 95°C for 30 s for initial denaturation, followed by 40 cycles of denaturation at 95°C for 5 s, annealing at 60°C for 34 s, and extension at 72°C for 40 s. At the end of each reaction, melting curve analysis was performed. *β-actin* was used as the reference gene and the 2^-ΔΔCT^ method was used to analyze the relative mRNA expressions (**Table 1**).

**Table 1:** Primer sequences used for real-time PCR (qPCR) analysis.

Gene	Forward primer	Reverse primer
*β-actin* *Col2* *Aggrecan*	5′-CCCGCGAGTACAACCTTCT-3′ 5′-CTCAAGTCGCTGAACAACCA-3′ 5′-GCAGACATTGATGAGTGCCTC-3′	5′-CGTCATCCATGGCGAACT-3′ 5′-GTCTCCGCTCTTCCACTCTG-3′ 5′-CTCACACAGGTCCCCTCTGT-3′
*Col10* *Mmp1*	5′-GATCATGGAGCTCACGGAAAA-3′ 5′-GGTGTGGTGTCTCACAGCTT-3′	5′-CCGTTCGATTCCGCATTG-3′ 5′-CGCTTTTCAACTTGCCTCCC-3′
*Mmp2*	5′-GATACCCCTTTGACGGTAAGGA-3′	5′-CCTTCTCCCAAGGTCCATAGC-3′
*Sox5* *Adamts9* *Adamts5* *Adamts4*	5′-GGGGAGACAGATGGAGAGGT-3′ 5′-TACAGGCAAAGGCTGGTCTC-3′ 5′-TGGAGTGTGTGGAGGGGATA-3′ 5′-TTCGCTGAGTAGATTCGTGGAG-3′	5′-GTGAGGCTTGTTGGGAAAAC-3′ 5′-CTCAGGTAGCAGGGATGGAC-3′ 5′-CGGACTTTTATGTGGGTTGC-3′ 5′-TGAGTCGTTCGGAGGGTTTAG-3′

### Statistical Analysis

Data were expressed as mean ± SD and subjected to one-way ANOVA, followed by Fisher’s least significant difference (LSD) comparison. All analyses were performed using an updated version of DPS software (Tang and Feng, 2007).

## Results

### Chemoprofiles of *Hsp* Extracts

**Figure 1:**
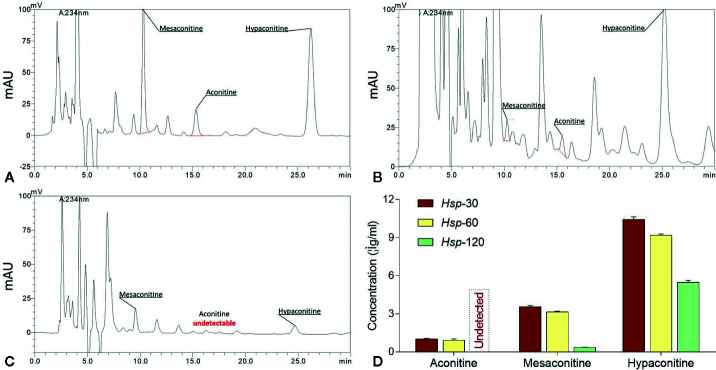
HPLC chromatograms of *Hsp*-30 **(A)**, *Hsp*-60 **(B)**, and *Hsp*-120 **(C)**, and concentrations of aconitine, mesaconitine, and hypaconitine in *Hsp*-30, *Hsp*-60, and *Hsp*-120 **(D)**.

The HPLC chromatogram comparison of toxic components in *Hsp*-30, *Hsp*-60, and *Hsp*-120 extracts were shown in **Figures 1A–C**. The retention time of aconitine, mesaconitine, and hypaconitine in all samples were 21.091 – 22.092 min, 11.162 – 11.816 min, and 44.705 – 47.280 min, respectively. There were no remarkable chromatographic differences in the peak time of *Hsp* samples with different decocting times. Nevertheless, upon decoction with water, the peak height of each compound decreased gradually in a time-dependent manner, and the peak of aconitine was even found disappeared after 120 min decoction. The concentrations of three toxic components in *Hsp*-30, *Hsp*-60, and *Hsp*-120 are shown in **Figure 1D**. The highest concentrations were found in *Hsp*-30, which attained 1.04 ± 0.03 μg/g for aconitine, 3.57 ± 0.07 μg/g for mesaconitine, 10.44 ± 0.20 μg/g for hypaconitine. Upon decoction with water, a stepwise decrease of each concentration was observed in different *Hsp* extracts with increasing decoction time. Especially when the decoction time attained 120 min, the concentrations of the three compounds reached the lowest level with undetected aconitine, 0.37 ± 0.01 μg/g of mesaconitine, and 5.47 ± 0.16 μg/g of hypaconitine. Thus, *Hsp*-120 was selected as d*Hsp* for the following animal experiment.

**Figure 2:**
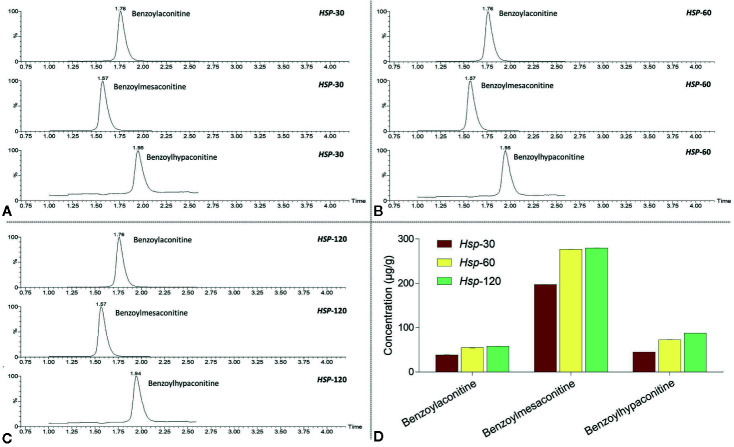
UPLC-MS chromatograms of *Hsp*-30 **(A)**, *Hsp*-60 **(B)**, and *Hsp*-120 **(C)**, and concentrations of benzoylaconitine, benzoylmesaconitine, and benzoylhypaconitine in *Hsp*-30, *Hsp*-60, and *Hsp*-120 **(D)**.

UPLC-MS was applied to identify and quantify the non-toxic derivatives of aconitines in *Hsp*-30, *Hsp*-60, and *Hsp*-120. As shown in **Figure 2**, benzoylaconitine, benzoylmesaconitine, and benzoylhypaconitine were all present in *Hsp*-30, *Hsp*-60, and *Hsp*-120, while their concentrations were increased with increasing decoction time. For example, benzoylaconitine was increased from 38.33 ± 0.44 μg/g in *Hsp*-30 to 58.08 ± 0.25 μg/g in *Hsp*-120, benzoylmesaconitine was increased from 197.20 ± 0.25 μg/g in *Hsp*-30 to 279.56 ± 0.36 μg/g in *Hsp*-120, and benzoylhypaconitine was increased from 44.90 ± 0.05 μg/g in *Hsp*-30 to 87.86 ± 0.09 μg/g in *Hsp*-120.

### Acute Toxicity of *Hsp* Extracts

**Figure 3:**
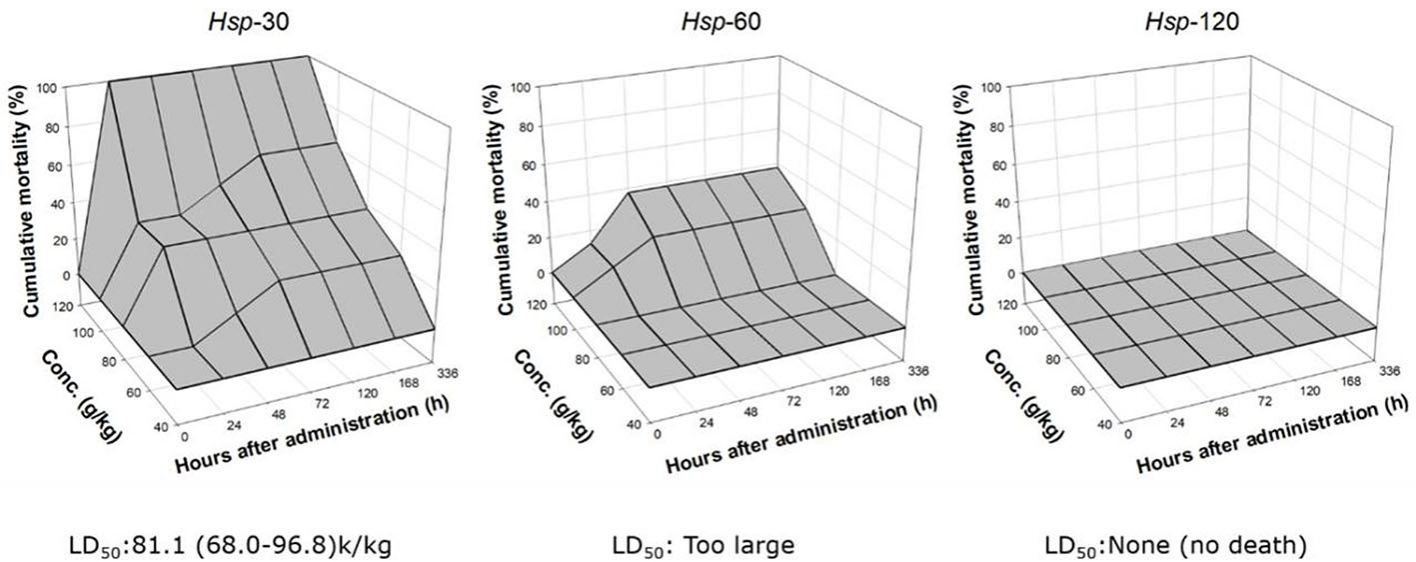
Cumulative mortalities of mice after oral administration of *Hsp*-30, *Hsp*-60, and *Hsp*-120 (g/kg) with LD_50_ value and associated 95% confidence limits.

Our preliminary experiment found a rational dose range of d*Hsp* being 40 to 120 g/kg for the acute toxicity test, since 120 g/kg almost reached the maximum saturated solubility. As shown in **Figure 3**, within 24 h, the death was initiated by *Hsp*-30 at 80 g/kg and *Hsp*-60 at 100 g/kg with mortalities of 37.5% and 12.5%, respectively. At the end, the mice exposed to *Hsp*-30 at 120 g/kg were all dead with LD_50_ of 81.1 (68.0 to 96.8) k/kg, indicating a concentration-dependent and time-dependent manner of toxicity. *Hsp*-60 showed lesser acute toxicity than *Hsp*-30, which caused final mortality of 37.5% at 120 g/kg. However, no death occurred under the treatment of *Hsp*-120 within the dose and time ranges, indicating a certain safety of *Hsp*-120.

### *In Vivo* Anti-OA Effect of d*Hsp*

**Figure 4:**
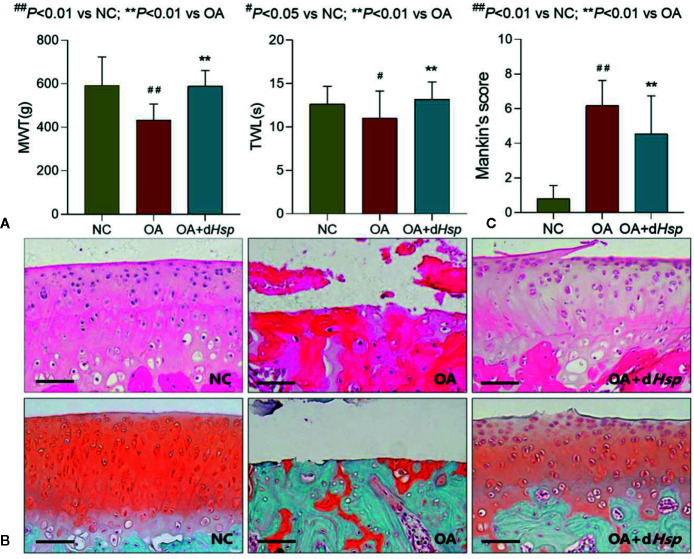
**(A)** MWT (g), TWL (s) results of the left and right hind paws of rats tested after final d*Hsp* treatment (28 d); **(B)** Histopathological observation (HE and Safranin-O staining); **(C)** Mankin’s scoring of rat knee joints at day 28 after d*Hsp* treatment. Values are presented as mean ± SD. ^#^ *P* < 0.05 vs. NC group; ^##^ *P*<0.01 vs. NC group; **P<0.01 vs. OA group. Scale bar = 100 μm.

MWT and TWL, respectively, reflect two behavioral responses: mechanical hyperalgesia and thermal hyperalgesia. As shown in **Figure 4A**, MWT and TWL were significantly decreased with OA modeling (both *P* < 0.05 versus NC group), while the abnormal levels were significantly restored by d*Hsp* after 28 days treatment (both *P* < 0.01 versus OA group). Histopathological results with HE and SO staining are shown in **Figure 4B**. Severe cartilage damage, characterized by loss of chondrocytes and disorganization of extracelluar matrix, was observed in the OA group with significant increase of Mankin’s score (**Figure 4C**, *P* < 0.01 versus NC group). As expected, the damaged phenotype was obviously relieved by d*Hsp* treatment with significant decrease of Mankin’s score (**Figure 4C**, *P* < 0.01 versus OA group), in which the chondrocytes and extracelluar matrix were protected.

### Molecular Actions of d*Hsp* on Cartilage

**Figure 5:**
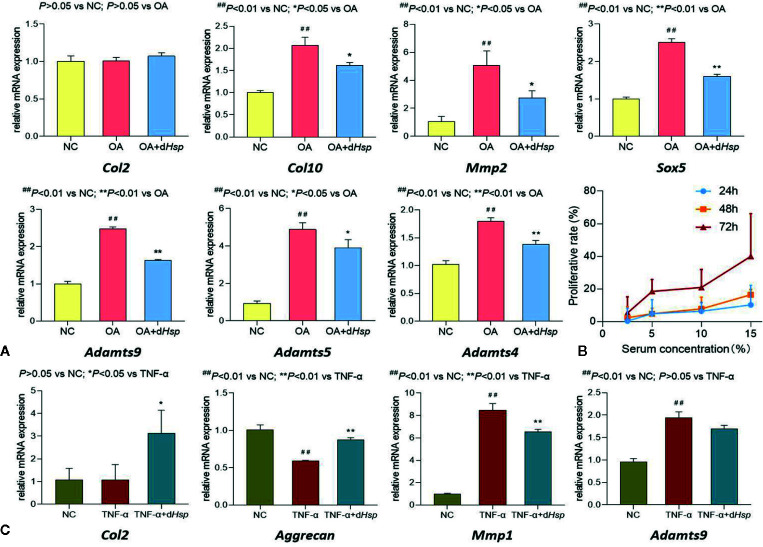
**(A)** Relative mRNA expressions of OA-related genes in cartilage tissue after oral administration of saline (NC group), saline (OA group), and 14 g/kg d*Hsp* (OA+ d*Hsp* group). **(B)** Cell viability of chondrocytes treated with d*Hsp-*containing serum for 24, 48, and 72 h. **(C)** Relative mRNA expressions of target genes in chondrocytes treated with TNF-α or TNF-α plus d*Hsp-*containing serum for 24 h. Values are presented as mean ± SD. ^##^P<0.01 vs. NC group; *P<0.05 or **P<0.01 vs. TNF-α group.

The molecular actions of d*Hsp* on the expression of OA-related genes in cartilage tissue were determined using qPCR assay. As shown in **Figure 5A**, MIA significantly up-regulated the expressions of *Col10*, *Mmp2*, *Sox5*, and *Adamts4/5/9*, as compared with that of NC group (all *P* < 0.01). After treatment of d*Hsp*, the abnormal expressions of those genes were significantly restored as compared with that of OA group (all *P* < 0.05 or 0.01). There was no obvious difference of *Col2* expression among all groups (*P* > 0.05).

### Cellular and Molecular Actions of d*Hsp*-Containing Serum on Chondrocytes

To mimic the *in vivo* behavior of d*Hsp*, d*Hsp-*containing serum was applied for *in vitro* experiments. The proliferative effect of d*Hsp-*containing serum on chondrocytes was assessed by MTT assay. As shown in **Figure 5B**, d*Hsp-*containing serum at concentration range from 2.5% to 15% significantly increased cell viability of chondrocytes. The proliferative rate was increased from 0.3% to 10.2% after 24 h treatment, from 2.3% to 16.4% after 48 h treatment, and from 5.2% to 40.0% after 72 h treatment. It indicated that d*Hsp-*containing serum induced proliferation of chondrocytes in concentration-dependent and time-dependent manner.

The molecular actions of d*Hsp-*containing serum on the expression of OA related genes in chondrocytes were determined by qPCR assay. The rat primary chondrocytes were pretreated with TNF-α to induce inflammatory response for mimicking the pathological condition of OA. As shown in **Figure 5C**, TNF-α significantly down-regulated the mRNA expression of *Aggrecan* and up-regulated the mRNA expressions of *Mmp1* and *Adamts9*, as compared with that of NC group (all *P* < 0.01). After 24 h treatment of d*Hsp-*containing serum, the abnormal expressions of those genes were all restored toward the normal levels, as compared with that of TNF-α group (all *P* < 0.05 except for *Adamts9*). Similar to the *in vivo* result, there was no obvious difference of *Col2* expression between NC and TNF-α groups (*P* > 0.05). Nevertheless, after 24 h treatment of d*Hsp-*containing serum, the *Col2* expression was significantly up-regulated as compared with that of TNF-α group (*P* < 0.05).

### Cellular and Molecular Actions of d*Hsp*-Contained Compounds on Chondrocytes

To further explore the potential mechanism of d*Hsp* on OA, cellular and molecular effects of d*Hsp*-contained alkaloids on chondrocytes were studied. MTT assay was conducted to evaluate the effect of aconitine, mesaconitine, hypaconitine, benzoylaconitine, benzoylmesaconitine, and benzoylhypaconitine on chondrocytes. For evaluating the contribution of each compound to d*Hsp*, the dose range for each compound was selected in accordance with their concentrations in d*Hsp*. For example, 5, 10, and 20 μg/ml were used as low, middle and high doses for aconitine, mesaconitine, hypaconitine, since the highest concentration of toxic aconitine in d*Hsp* was 5.47 ± 0.16 μg/ml (hypaconitine). Moreover, 30, 60, and 120 μg/ml were used as low, middle and high doses for benzoylaconitine, 140, 280, and 560 μg/ml were used as low, middle and high doses for benzoylmesaconitine, and 45, 90, and 180 μg/ml were used as low, middle and high doses for benzoylhypaconitine, in which their middle doses were similar to their concentrations in d*Hsp*.

**Figure 6:**
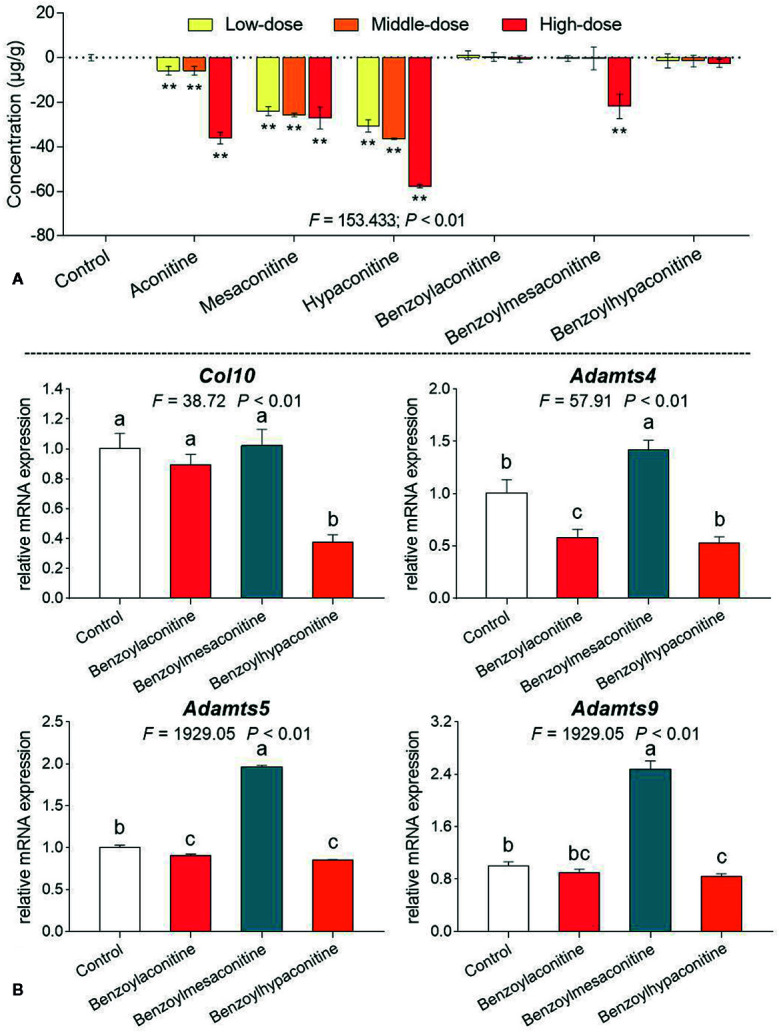
Cell viability of chondrocytes with treatments of aconitine, mesaconitine, hypaconitine, benzoylaconitine, benzoylmesaconitine, and benzoylhypaconitine **(A)** and relative mRNA expressions of OA-related genes in chondrocytes with treatments of benzoylaconitine, benzoylmesaconitine, and benzoylhypaconitine **(B)**. Values are presented as mean ± SD. By means of LSD multiple comparisons, data with same lowercase letter indicate no significant difference between each other, while data with different letters indicate significant difference with each other. **P<0.01 vs. control group.

As shown in **Figure 6A**, after 24 h treatment, aconitine, mesaconitine, and hypaconitine exerted significant inhibitory effects on chondrocytes at their dose ranges (all *P* < 0.01), indicating cytotoxicity of these compounds against chondrocytes. Besides, benzoylaconitine and benzoylhypaconitine at their dose ranges had non-toxic effect on chondrocytes, while benzoylmesaconitine at high dose exerted significant inhibitory effect (*P* < 0.01), suggesting that these compounds at their dose ranges, except benzoylmesaconitine at high dose, may contribute to the therapeutic effect of d*Hsp*. Thus, these three compounds were selected for the following qPCR assay. As shown in **Figure 6B**, as compared with control group, benzoylaconitine at middle dose significantly down-regulated the expressions of *Adamts4* and *Adamts5* with slight down-regulatory effect on *Col10* and *Adamts9*, while benzoylhypaconitine at middle dose significantly down-regulated the expressions of *Col10*, *Adamts4*, *Adamts5*, and *Adamts9*, suggesting these compounds as therapeutic components of d*Hsp*. However, benzoylmesaconitine at middle dose significantly up-regulated the expressions of *Adamts4*, *Adamts5*, and *Adamts9* with slight up-regulatory effect on *Col10*, indicating a negative contribution of this compound to d*Hsp*.

## Discussion

Joint impairment-associated pain is the key clinical feature of OA, mainly caused by synovial neurogenic inflammation and subchondral nerve damage (Neogi et al., 2016). With the onset of joint impairment, pain occurs and progressively disables the joint capacity and movement in OA patients. Thus, it is very important to reach pain relief during OA therapy and to employ specific preclinical model of OA that can be used to evaluate not only disease-modifying effects but also analgesic activity. MIA-induced rat model is a minimally invasive, rapidly developed, and reproducible OA model, which causes histomorphological and functional joint impairment as well as pain behavior similar to human OA (Pitcher et al., 2016). MIA injected in articular cavity disrupts cartilage glycolysis and chondrocyte metabolism by inhibition of glyceraldehye-3-phosphate dehydrogenase, thereby inducing chondrocyte death, cartilage destruction and subchondral bone exposure (Lampropoulou-Adamidou et al., 2014). Subchondral bone is abundantly innervated and could potentially be a source of OA pain (Philpott et al., 2017). In addition, the severity of OA progression in this model can be controlled by MIA in a dose-dependent manner, therefore, the model of OA induced by MIA has become one of the most popular models to research the pain course and intervention effect of OA (Ogbonna et al., 2013; Chin et al., 2019).

As a commonly used TCM product, *Hsp* has attracted much attention for its therapeutic efficacy. However, it remains potentially toxic to patients (Liu et al., 2016). Long-time decoction is a traditional and rational method for detoxifying *Hsp*, the principle of which is that acetyl group at C_8_ and benzoyl group at C_14_ of DDAs are hydrolyzed under the action of water and heat, thereby reducing its content to obtain less toxic monoester-diterpenoid alkaloids and non-toxic non-esterified diterpenoid alkaloids (Yang et al., 2018). Up to now, it remains uncertain, how long time should be used and what effect it has. Previously, by means of long-time (120 min) decoction, we successfully removed the acute toxicity of *Bai-fu-pian* (*Bfp*), another kind of *Fu-zi*, and demonstrated unaltered therapeutic efficacy of detoxified *Bfp* (d*Bfp*) on rheumatoid arthritis (Tong et al., 2013). It suggests that 120 min decoction might be effective in detoxifying *Hsp*. Considering the difference between *Bfp* (with no peel) and *Hsp* (with peel) as well as the more common use of *Hsp* in clinic, it is necessary to demonstrate whether long-time (120 min) decoction is also effective in detoxifying *Hsp* without alteration of therapeutic efficacy. Therefore, this study extracted *Hsp* with gradient decoction times (30, 60, and 120 min) and studied their acute toxicity and chemoprofiles. The results showed that, with increasing decocting time, the toxicity and concentrations of toxic alkaloids of *Hsp* were time-dependently decreased and the concentration of non-toxic alkaloid derivatives increased (**Figures 1** **–** **3**). *Hsp* with 120 min decoction was found to contain minimal levels of toxic components (**Figure 1**) and maximal levels of non-toxic derivatives (**Figure 2**). Also, it was non-toxic within the maximal dose range (**Figure 3**). In fact, 120 min is the upper time limit for the decoction, since more than 120 min would be too long to be practical. Thus, we defined the 120 min decocted *Hsp* as detoxicated *Hsp* (d*Hsp*). Afterwards, an animal model of OA was employed to evaluate the therapeutic effect of d*Hsp*. After 28 day treatment, the analgesic effect of d*Hsp* was determined by assessments of mechanical and thermal sensitivity (**Figure 4A**). Meanwhile, the histopathological evidence combined with Mankin’s grading analysis exhibited the chondroprotective effect of d*Hsp*. To further clarify the molecular actions of d*Hsp*, qPCR assay was conducted on both cartilage and chondrocytes. d*Hsp* significantly restored the abnormal expressions of *Col10*, *Mmp2*, *Sox5*, and *Adamts4/5/9* and up-regulated the *Col2* expression in damaged cartilage tissue. It also significantly restored the abnormal expressions of *Aggrecan*, *Mmp1* and *Adamts9* and up-regulated the *Col2* expression in TNF-α treated chondrocytes (**Figures 5A, C**). Moreover, the results of MTT assay showed that d*Hsp* significantly increased the cell viability of chondrocytes (**Figure 5B**). Further cellular and molecular assays showed that, among the six alkaloid components of *Hsp*, toxic alkaloids (aconitine, mesaconitine, and hypaconitine) were cytotoxic to chondrocytes, while benzoylaconitine and benzoylhypaconitine exhibited molecular actions similar to d*Hsp* (**Figure 6**). The result suggests that benzoylaconitine and benzoylhypaconitine are therapeutic components of d*Hsp*, which give positive contribution to the anti-OA effect of d*Hsp*.

The destruction of cartilage in the process of OA is closely related to the degradation of cartilage extracellular matrix (ECM) (Guilak et al., 2018). The ECM of cartilage is mainly composed of collagens and proteoglycan. Col2 is the main collagen in the ECM and plays an important role in the metabolism and stability of cartilage. Col10 is another collagen existing rarely in the healthy cartilage but mainly in the degenerative cartilage. It is highly expressed by hypertrophic chondrocytes as a marker of hypertrophic regeneration. In sections of human osteoarthritic cartilage, Col10 was found around hypertrophic chondrocyte clusters in the deep zone close to tidemark (He et al., 2019). Besides the collagens, aggrecan, known as a cartilage-specific proteoglycan core protein, is another crucial component of cartilage ECM (Hu et al., 2019). In the OA state, aggrecan was significantly degraded in the cartilage, and the expression of aggrecan was inversely related to the OA progression (Eid et al., 2006). In contrary, MMPs and ADAMTSs are enzymes positively related to the OA progression, which can destroy the integrity and function of cartilage by hydrolyzing the ECM (Malemud, 2019). Of these, MMP1 exerts the strongest degradation effect on Col2 as a collagenase produced by synovial cells (Mehana et al., 2019). MMP2, known as gelatinase A, is also an important factor in the pathogenesis of OA (Zeng et al., 2015). Among the members of ADAMTSs, ADAMTS9, ADAMTS 5 and ADAMTS 4 have been shown to greatly induce degradation of cartilage aggrecan and fibrosis of collagen, resulting in the loss of cartilage compression strength and eventual occurrence of OA (J. Bondeson et al., 2008; Ohtsuki et al., 2019). Sox5 acts as a transcription factor associated with chondrogenesis. It is a highly expressed in synovium with inflammation, and the degree of cartilage destruction can be significantly reversed when the expression of Sox5 is silenced (Feng et al., 2016). In this study, we found that the molecular actions of d*Hsp* were mediated by the regulation of gene expressions of *Col2*, *Aggrecan*, *Col10*, *Mmp1*, *Mmp2*, *Adamts4/5/9*, and *Sox5* in cartilage tissue and chondrocytes, suggesting a mechanism of d*Hsp* in association with the inhibition of ECM degradation and chondrocyte hypertrophy.

## Conclusions

This is the first study reported that d*Hsp* has anti-OA effect independent of its toxicity, which provides a safe candidate for TCM formulation in OA therapy. So far, there are two issues warranting further investigation. Firstly, d*Hsp* has not completely reversed the OA histopathological changes in this study, since many chondrocytes remained lost on the cartilage. It might be due to the excessive severity of our MIA model. More OA models with moderate severity are needed for further evaluation the d*Hsp*′s effect. Secondly, although the representative alkaloids in d*Hsp* have been analyzed by this study, the roles of other components in d*Hsp* remain unclear and need to be explored in future.

## Data Availability Statement

The datasets generated during and/or analysed during the current study are available from the corresponding author on reasonable request..

## Ethics Statement

The animal study was reviewed and approved by the Medical Norms and Ethics Committee of Zhejiang Chinese Medical University.

## Author Contributions

LZha performed and funded the main work for revision; TL contributed to the main work of this study. RW performed the chemical analysis and wrote the paper. JX performed the MTT and qPCR assays for revision. LZho and LY contributed to the molecular experiment. ZH performed the UPLC-MS assay. HL participated in cellular experiments. FL contributed to the design of this study. WD and HW designed and funded this study. LS designed this study and finalized the data analysis. PT provided research ideas. SZ participated in the data analysis. TE improved the experimental design and the writing of this paper. All authors contributed to the article and approved the submitted version.

## Funding

This study was supported by the National Natural Science Foundation of China (Grant No. 81774331, 81873049, 81804125), the Zhejiang Provincial Natural Science Foundation of China (Grant No. LY18H270004), the Zhejiang Provincial Science and Technology Project of Traditional Chinese Medicine of China (Grant No. 2016ZZ011), the Zhejiang Provincial Key Construction University Superiority Characteristic Discipline (Traditional Chinese Pharmacology) Opening Foundation of China (Grant No. ZYX2018006), and the Ningbo Natural Science Foundation (2014A610277).

## Conflict of Interest 

The authors declare that the research was conducted in the absence of any commercial or financial relationships that could be construed as a potential conflict of interest.

## Abbreviations

DDAs, diester-diterpenoid alkaloids; d*Hsp*, detoxicated *Hsp*; DMSO, dimethyl sulfoxide; ECM, extracellular matrix; FBS, fetal bovine serum; HE, hematoxylin and eosin; *Hsp*, *Hei-shun-pian*; IMDM, Iscove’s modified Dulbecco’s medium; LSD, least significant difference; MIA, mono-iodoacetate; MWT, mechanical withdrawal threshold; OA, osteoarthritis; PCR, real time polymerase chain reaction; SO, safranin-O; TCM, traditional Chinese medicine; TWL, thermal withdrawal latency.
